# Trends in the Prevalence of Chronic Medication Use Among Children in Israel Between 2010 and 2019: Protocol for a Retrospective Cohort Study

**DOI:** 10.2196/36756

**Published:** 2022-08-05

**Authors:** Yair Sadaka, Dana Horwitz, Leor Wolff, Tomer Sela, Joseph Meyerovitch, Assaf Peleg, Eitan Bachmat, Arriel Benis

**Affiliations:** 1 Neuro-Developmental Research Centre Beer Sheva Mental Health Centre Ministry of Health Beer Sheva Israel; 2 Faculty of Health Sciences Ben-Gurion University of the Negev Beer Shevs Israel; 3 Online and IT Division Clalit Health Services Tel-Aviv Israel; 4 Community Division Clalit Health Services Tel-Aviv Israel; 5 Institute for Endocrinology and Diabetes National Center for Childhood Diabetes Schneider Children's Medical Center of Israel Petah Tikva Israel; 6 Sackler Faculty of Medicine Tel Aviv University Tel Aviv Israel; 7 Department of Computer Science Ben-Gurion University of the Negev Beer Sheva Israel; 8 Faculty of Industrial Engineering and Technology Management Holon Institute of Technology Holon Israel; 9 Faculty of Digital Technologies in Medicine Holon Institute of Technology Holon Israel

**Keywords:** psychotropic drugs, central nervous system stimulants, mental health, medication therapy management, drug prescriptions, attention deficit hyperactivity disorder, ADHD, Israel, children, data mining, machine learning, electronic medical records, pediatrics, chronic disease, epidemiology

## Abstract

**Background:**

Prescription of psychostimulants has significantly increased in most countries worldwide for both preschool and school-aged children. Understanding the trends of chronic medication use among children in different age groups and from different sociodemographic backgrounds is essential. It is essential to distinguish between selected therapy areas to help decision-makers evaluate not only the relevant expected medication costs but also the specific services related to these areas.

**Objective:**

This study will analyze differences in trends regarding medications considered psychobehavioral treatments and medications considered nonpsychobehavioral treatments and will identify risk factors and predictors for chronic medication use among children.

**Methods:**

This is a retrospective study. Data will be extracted from the Clalit Health Services data warehouse. For each year between 2010 and 2019, there are approximately 1,500,000 children aged 0-18 years. All medication classes will be identiﬁed using the Anatomical Therapeutic Chemical code. A time-trend analysis will be performed to investigate if there is a significant difference between the trends of children’s psychobehavioral and nonpsychobehavioral medication prescriptions. A logistic regression combined with machine learning models will be developed to identify variables that may increase the risk for specific chronic medication types and identify children likely to get such treatment.

**Results:**

The project was funded in 2019. Data analysis is currently underway, and the results are expected to be submitted for publication in 2022. Understanding trends regarding medications considered psychobehavioral treatments and medications considered nonpsychobehavioral treatments will support the identification of risk factors and predictors for chronic medication use among children.

**Conclusions:**

Analyzing the response of the patient (and their parents or caregivers) population over time will hopefully help improve policies for prescriptions and follow-up of chronic treatments in children.

**International Registered Report Identifier (IRRID):**

DERR1-10.2196/36756

## Introduction

### Background

The rising health expenditure on medications, especially chronic medications, accounts for a large share of total health care spending and is a major financial issue. US prescription drug spending as a share of health expenditures was estimated to be between 9.8% and 16.8% by different estimators in 2016 [1]. Over the last decade, there has been a growth in the amounts of chronic medications used by patients with chronic diseases in the United States. When evaluating dispensed prescriptions in different therapy areas in the United States, the most significant growth between 2012 and 2017 is observed in medications prescribed for mental health conditions [2]. Similarly, in Israel, health care–related expenditures are also increasing over time, mainly involving private financing such as private insurance [3]. For example, looking at mental health conditions, the incidence of first prescription of stimulant medication for attention-deficit/hyperactivity disorder (ADHD) is growing among children in Israel year-over-year [4,5].

Understanding medication use among patients with chronic diseases is critical to evaluate health care costs better and allocate funds more effectively. Due to the global lack of data in the literature, and more particularly related to Israel in recent years, there is a need to study these issues, with a specific focus on children [6]. Although children more often have acute, short-term illnesses including upper respiratory tract and ear infections, gastroenteritis, and injury-related complaints, a growing body of literature has identified an increased risk for chronic conditions such as obesity [7], asthma [8], type 1 and type 2 diabetes mellitus [9], and ADHD and other psychiatric conditions [10-12].

One challenge to children and youth health is the wide use of psychobehavioral treatments for disruptive behavior disorders [13-16]. Indeed, there is an increasing trend of prescribing psychotropic medications in the pediatric population [17-19]. These psychobehavioral treatments are psycholeptics and psychoanaleptics, which are classified in the Anatomical Therapeutic Chemical (ATC) classification system as codes N05 and N06, respectively [20]. One of the main indications for prescribing psychotropic medications in children is the psychostimulant treatment for ADHD [4,5,21]. Prescription of psychostimulants has significantly increased in most countries worldwide for both preschool and school-aged children [22,23], as well as in Israel, where up to 10% of children have been prescribed psychostimulants [4,5,21,22].

### Aims and Objectives

Studying trends in chronic medication use among children in different age groups and from varying sociodemographic backgrounds and distinguishing selected therapy areas may help decision-makers evaluate the relevant expected medication costs and the specific services related to these areas. This study aims to analyze trends in chronic medication use and identify risk factors for such treatments.

## Methods

### Data Acquisition

This is a retrospective cohort study. The analysis will be done on data extracted from the Clalit Health Services (Clalit) data warehouse. Clalit, the largest health care management organization in Israel, insures 52% of the Israeli population (approximately 4.6 million members), operates 14 hospitals, and manages over 1300 primary care clinics with a network of pharmacies and dental clinics.

The electronic medical records of approximately 1,500,000 children aged 0-18 years old whom Clalit insured each year between 2010-2019 will be analyzed. It is important to highlight that children are those aged between 0 and 18 years according to the current Israeli regulation and the national medical services provided in Israel and thus the same applies to the “pediatric” databases we have access to for our study.

The Clalit data warehouse stores sociodemographic data (date and country of birth, immigration status, gender, marital status of parents, clinic location–based socioeconomic status, and ethnicity) and medical data. Clinical data are collected from continuous real-time inputs from physicians and health service providers from the community and hospital settings and include medical diagnoses, laboratory data, prescription renewal, medications dispensed, clinic and emergency room visits, and consultation appointments with specialists. All relevant information includes a patient identifier, which allows compiling all data relevant to a specific patient into a single record.

In Israel, like in much of the world, a prescription can be valid for a few months (3-12 generally), but the prescription can only be filled by the pharmacist one month (28-30 days) at a time. If a patient purchases a treatment for one month or less, they cannot be defined as a patient with chronic treatment; rather, chronic medical treatment can be defined as a documented purchased prescription for 60 days or more within a year, denoted as “purchased chronic medication” for that year. This definition is valid for adults but, to our knowledge, no definition exists for children. Thus, we will consider it relevant for our study and use the same definition [24,25]. Therefore, it seems preferable to look at recurrent delivery of medications rather than diagnosis, even though the latter may be available in the Clalit data warehouse. Indeed, it is well known that finding and characterizing diseases can be accomplished by integrating different parameters such as biological tests, clinical diagnoses, and medication prescriptions and purchases [26]. However, as this research aims to highlight the prevalence of chronic medication, the diagnosis itself is less critical than the use of medication over time. Additionally, it is essential to point out that the Israeli health system regulation changed in 2015—only since then have non–mental health professionals been able to get diagnosis and medication prescription information about a patient [27]. Thus, medication purchase is the better option for following chronic medication trends.

The chronic medications will be classified using the ATC code [20]. We will further analyze different classes of medications with specific attention to psychobehavioral treatment if classified by the ATC as N05 (psycholeptics) or N06 (psychoanaleptics).

We will further collect, according to the updated approval of the data extraction and ethics committees, sociodemographic variables (ie, date of birth, gender, immigration status, ethnicity, socioeconomic status, geographical region of residency, health care management organization–specific insurance type) and clinical variables (ie, diagnosed medical conditions such as comorbid diagnoses, health services consumption, and lab test results when relevant) for further analysis.

### Ethics Approval

Ethics approval was granted by the ethical committee of the Soroka Medical Center of Clalit (0011-18-MHC; February 3, 2019).

### Data Inclusion and Exclusion Criteria

Children are members of Clalit with at least one of their parents. Clalit members between January 2010 and December 2019 aged 0-18 years during that period will be included in the study. However, individuals who were Clalit members for less than one year between January 2010 and December 2019 will be excluded. Members that joined (not newborns) between January 2010 and December 2019 were included, with their related data from the date they joined if they were members for more than one year. Members that left (for any reason) between January 2010 and December 2019 were included if they were members for more than one year.

### Data Preprocessing

Several studies will be conducted based on the data that will be retrieved.

### Analysis

#### Descriptive Analysis and Trend Analysis

We will calculate the incidence and prevalence of prescribed and purchased medications for different ATC groups between 2010 and 2019. Trend estimation is a statistical technique to aid the interpretation of data [28]. When a series of measurements of a process is treated as a time series, trend estimation can be used to make and justify statements about tendencies in the data by relating the measurements to the times at which they occurred. These models can then be used to describe the behavior of the observed data without explaining it. Once data are available, an appropriate trend analysis will be conducted for the prevalence and incidence of psychobehavioral and nonpsychobehavioral medication prescriptions. We will further evaluate whether there is a significant difference between psychobehavioral and nonpsychobehavioral medication prescription trends.

#### Logistic Regression Models

We will compare demographic characteristics between the study groups (children who received different classes of medications for chronic diseases vs children who did not receive such medications) using *t* tests and Fisher exact χ^2^ tests for categorical variables based on the normal distribution and variable characteristics. Categorical data will be shown in counts and percentages. Data on continuous variables with normal distribution will be presented as the mean and 95% confidence interval. We will use multivariable logistic regression and a lasso regression [29] to identify variables that may increase the risk for specific types of chronic medication treatment using all available sociodemographic and clinical attributes for disentangling the effects of moderately correlated variables. Moreover, if we detect some medications with too few children treated, we will deal with this issue by considering using a zero-inflated Poisson regression model. It is used to model count data that has an excess of zero counts, or in the context of this study, medication treatments that are not prescribed and delivered to many children (regarding the different attributes defining them).

#### Hierarchical Clustering Analysis

In addition to the “classical” statistical analysis described above, further data analysis will combine the previous methods with a data mining approach and machine learning algorithms.

We will mainly focus on hierarchical clustering and information visualization (ie, heatmaps) [5,30]. This will facilitate the analysis of the incidence and prevalence trends of prescriptions and the purchase of the different investigated medications, with regard to the collected attributes and their predefined categories (eg, age groups, socioeconomic status). In other words, the clustering analysis and its visualization as heatmaps give snapshots of medication prescriptions and purchases (as a proxy of consumption).

The incidence and the prevalence will be normalized for each ATC class.

We will compute a Euclidean distance matrix between the values of each of the normalized epidemiological measures (ie, incidence, prevalence) for the sociodemographic and clinical attributes categories. For example, we will compute a distance matrix of the normalized incidence for the prescribed medications (ie, cells of the matrix), each of the sociodemographic categories, diagnosed medical conditions, and health services consumption-related attributes (ie, rows and columns of the matrix).

Then, the heatmap will consist of a set of ordered columns according to the similarity of the attributes computed by hierarchical clustering. The heatmap’s rows will consist of the values (ie, reformulated as a color gradient) of the incidence of the prescription or the purchase over the years.

We will build the following heatmaps of the overall medications over time:

- the overall prescriptions
- the overall purchases
- the psycholeptics and psychoanaleptics (prescriptions as a whole and purchases as a whole)
- the psycholeptics (prescriptions as a whole and purchases as a whole)
- the psychoanaleptics (prescriptions as a whole and purchases as a whole)

The use of the hierarchical clustering approach for drawing heatmaps for each epidemiological metric (ie, incidence, prevalence) and for each step of the medication consumption (ie, prescription, purchase) allows showing similarities and changes over time of the different attributes (ie, sociodemographics and clinical data) related to the patients of the analyzed population.

## Results

This project was funded in 2019, and the research project is scheduled to be completed in 2022.

A preliminary analysis has been performed on data from 2010 and 2015 that included 1,297,535 children aged 0-18 years in 2010 and 1,472,190 in 2015. Overall, we found that chronic medication prescription and purchase are more prevalent for children aged 0-1 years and 16-18 years. Additionally, we observed an increase in purchased prescriptions of chronic medications for children aged 11-15 years and for older children aged 16-18 years.

Additional analyses are underway and will investigate the data more deeply by looking at the differences between girls and boys from different groups (eg, secular Jews, Orthodox Jews, and Arabs) as defined by the Israeli Central Bureau of Statistics.

Moreover, logistic regression models and hierarchical clustering will be processed like their interpretation during the second quadrimester (April to August) of 2022.

## Discussion

### Overview

This research protocol deals with understanding trends regarding medications considered psychobehavioral treatments and medications considered nonpsychobehavioral treatments. This will support the identification of risk factors and predictors for chronic medication use among children. This knowledge will help the health care system evaluate the expected medication costs and allocate funds more effectively. The study hypothesis is that there will be a gradual increase in chronic medication use over the years. This trend will be more significant for psychobehavioral treatments.

### Expected Results and Future Directions

In Israel, an analysis of chronic medication use among children between 2010 and 2019 can reveal long-term prescription and treatment adherence patterns. Over time, analyzing the response of the patient (their parents or caregivers) population will hopefully help improve the policies for the prescriptions and the follow-up of chronic treatments in children.

### Strengths and Limitations

Recent chronic medication purchase trends were not extensively studied in the Israeli pediatric population.

Moreover, Clalit insured and provided medical services to approximately 4.7 million patients in 2021 and is the largest health care provider in Israel with one of the world’s largest medical data warehouses. The data available span all treatment providers, including hospitals and emergency units.

Nevertheless, this study is limited to Israel, and the overall ethnic distribution of the Clalit population does not fully reflect the overall Israeli demographic composition. The Clalit members comprise, in comparison with the general Israeli population, a higher proportion of Arabs, a lower proportion of ultra-Orthodox members, and globally a higher proportion of members having a low socioeconomic status [30].

## Abbreviations

ADHD: attention-deficit/hyperactivity disorder
ATC: Anatomical Therapeutic Chemical

## Data Availability

The data sets generated and analyzed during this study are not publicly available due to local regulation.
